# A comprehensive interventional program to improve the sexual function of women with endometriosis: a mixed-methods protocol study

**DOI:** 10.1186/s12978-024-01759-4

**Published:** 2024-02-27

**Authors:** Maryam Heidari Sarvestani, Mahnaz Noroozi, Maryam Hashemi, Firoozeh Mostafavi

**Affiliations:** 1 Student Research Committee, School of Nursing and Midwifery, Isfahan University of Medical Sciences, Isfahan, Iran; 2 Department of Midwifery and Reproductive Health, School of Nursing and Midwifery, Isfahan University of Medical Sciences, Isfahan, Iran; 3 Department of Obstetrics and Gynecology, School of Medicine, Isfahan University of Medical Sciences, Isfahan, Iran; 4 Department of Health Education and Promotion, School of Health, Isfahan University of Medical Sciences, Isfahan, Iran

**Keywords:** Sexual function, Endometriosis, Women, Mixed methods study, Iran

## Abstract

**Background:**

Endometriosis is a chronic disease affecting 6–10% of women worldwide. Sexual dysfunction has been reported in a significant percentage of these patients. Thus, the present study will be conducted to design, implement, and determine the effectiveness of an interventional program to improve the sexual function of women with endometriosis.

**Materials and methods:**

This mixed-methods study will be carried out in three phases with a sequential exploratory approach. In the first phase (qualitative study) participants will be selected by purposive sampling in Isfahan, Iran. The data will be collected through in-depth interviews and field notes and analyzed using conventional content analysis. The interventional program will be designed in the second phase based on the results of the qualitative study and literature review and using the Delphi method and panel of experts. The interventional program will be implemented at the individual level in the third phase to investigate its effect on improving women’s sexual function. This phase includes quasi-experimental research, in which the pre- and post-intervention data will be collected from the intervention and control groups using the FSFI questionnaire and analyzed by descriptive and inferential statistical methods. Ultimately, a suitable interventional program will be presented by combining the data obtained in the qualitative and quantitative phases of the research.

**Conclusion:**

Conducting the present study, along with the design and implementation of an appropriate, native, and culturally sensitive interventional program, can contribute to improving the sexual function of women with endometriosis and enhancing the quality of sexual relations between couples.

## Abstract (plain language summary)

Endometriosis is a chronic disease in women of reproductive age. The negative impact of endometriosis on intimate relationships and sexual function has been reported in a significant number of women with this disease. The results of this study offer a rich source of information for the required interventions to promote the sexual and reproductive health of women with endometriosis. This study is a sequential exploratory (qualitative–quantitative) mixed methods design that consists of three consecutive phases. In this study, following a qualitative approach, the researchers will explain the experiences of women with endometriosis of sexual function and the appropriate strategies to improve the sexual function of these women. In the second phase, the researcher will design an appropriate interventional program for to improving the sexual function of women with endometriosis by using the results of the qualitative phase and literature reviews. The purposed interventional program is designed using the Delphi method and panel of experts and will be finalized for execution. In the third stage, the effectiveness of interventional program on the sexual function of women with endometriosis will be investigated in a quasi-experimental study. Therefore, it is expected that conducting a mixed method study by presenting an interventional culturally sensitive program can contribute to improving the sexual function of women with endometriosis and enhancing the quality of sexual relations between couples.

## Background

Endometriosis is a chronic and often progressive disease affecting 6–10% of reproductive-age women worldwide, which is defined as the presence of endometrial tissue outside the uterus [1–4]. Clinical symptoms of endometriosis include dysmenorrhea, chronic pelvic pain, dyspareunia, backache, dyschezia, dysuria, and reduced fertility [1, 5]. According to the World Endometriosis Research Foundation, 47% of women with endometriosis have painful sex, and almost one out of every two women has sexual dysfunction [6]. In these patients, dyspareunia is associated with other forms of sexual dysfunction, such as decreased sexual desire, decreased lubrication, arousal problems, and orgasm disorders. All these disorders often coexist, reducing overall sexual satisfaction and increasing the risk of developing vaginismus and couple relationship disorders [6–8]. These women may also suffer from low self-esteem, psychological distress, stress, anxiety, depression, poor social support, and guilt due to limited sexual performance [9, 10]. Depression is related to sexual quality of life disorders, including sexual desire, arousal, sexual acceptance and understanding, and orgasmic functions [3]. Over the years, the primary goal of endometriosis treatment has been to reduce or eliminate pain in women, traditionally focusing on surgery and medical treatment [11]. Previously conducted studies have investigated the effect of surgical treatments, especially laparoscopy, mainly focusing on one aspect of sexual dysfunction, namely dyspareunia, and failing to consider the overall female sexual function [7, 12]. However, although surgical and pharmacological treatment options may improve sexual function in affected women, they do not necessarily lead to definitive and long-term solutions to female sexual dysfunction [5]. Thus, alleviation of clinical symptoms, attention to sexual life, and enhancement of these patients’ overall quality of life is among the main issues requiring management [12, 13].

Developing health promotion programs, especially on culturally sensitive issues such as sexual concerns, and paying attention to socially accepted cultures and customs can increase the effectiveness of the programs. Thus, an in-depth study should be carried out to improve the sexual function of women with endometriosis to design and implement effective local interventions. This mixed-methods study will be conducted to design, implement, and determine the effectiveness of an interventional program to improve the sexual function of women with endometriosis.

## Materials and methods

**Fig. 1:**
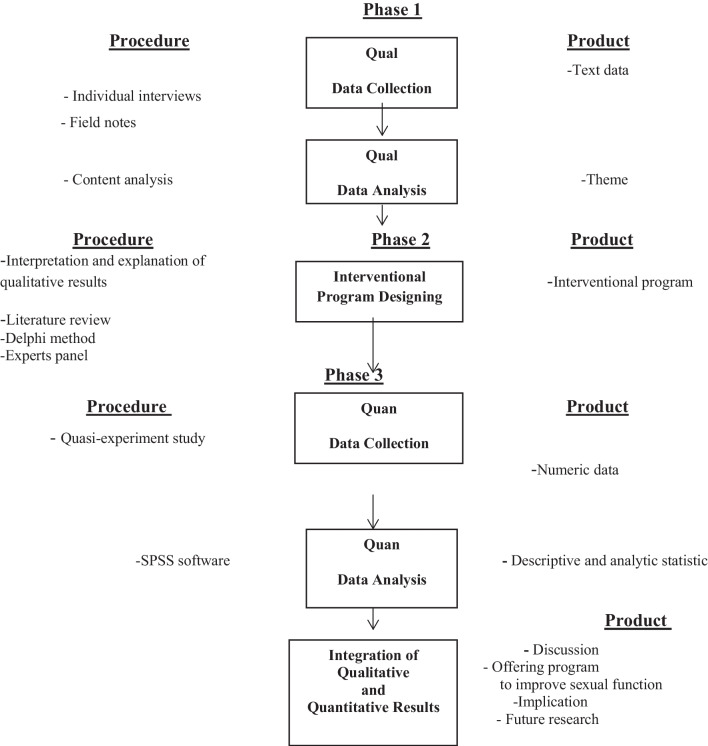
Study visual diagram

This study is an exploratory mixed-methods study with a sequential design (qualitative-quantitative) consisting of 3 consecutive phases. A qualitative study will be conducted in the first phase, followed by designing an interventional program in the second phase to improve the sexual function of women with endometriosis based on the qualitative study and literature review. The solutions obtained will be then prioritized using the modified Delphi panel method. The second phase also includes compiling the draft of the interventional program to improve the sexual function of women with endometriosis, making the necessary modifications in the draft, and preparing the final version of the interventional program. In the third phase, this program will be implemented and evaluated through a quasi-experimental study to check its effectiveness (Fig. 1).

### Phase I: qualitative study

#### Objectives

- Explaining the experiences of women with endometriosis of sexual function
- Explaining the appropriate strategies to improve the sexual function of women with endometriosis

This phase will be designed based on the answers to the following questions:

- What experiences do women with endometriosis report regarding their sexual relations?
- What are the appropriate solutions to improve the sexual function of women with endometriosis?

The qualitative content analysis method will be used in this phase.

*Participants* The participants will be women with endometriosis and those who have experience in providing health services to women with endometriosis, including midwives, obstetricians, reproductive health specialists, psychiatrists, and psychologists in Isfahan, Iran.

*Sampling method* The participants will be selected using purposive sampling, with an emphasis on the maximum variety in terms of age, occupation, education, economic status, length of marriage, number of pregnancies and deliveries, type of pharmacological treatment, and the time since the diagnosis of the disease. Health service providers will also be selected with the maximum variety in terms of their work experience.

#### Inclusion criteria for women with endometriosis

- Iranian nationality;
- Able to communicate and share experiences;
- At the reproductive age (19 to 45 years);
- Elementary and higher education;
- Married and sexually active;
- The only wife of her husband;
- Endometriosis diagnosis by a gynecologist;
- No pregnancy or breastfeeding;
- Not receiving treatment with assisted reproductive techniques (ART);
- No history of known psychological disorders requiring medication;
- No history of drug and alcohol abuse in the woman and her husband;
- The absence of premature ejaculation or impotence in husbands, according to the individual’s own report;
- Absence of surgical history in the participants and their spouses, such as prostatectomy, mastectomy, and other breast surgeries (through questions and document reviews);
- No history of surgery due to endometriosis;
- No history of stressful events in the last 6 months, such as the death of parents, etc.;
- No diseases that affect the sexual function of participants and their spouses, including systemic diseases such as liver, renal, and lung failure, cardiovascular diseases, cancers, ulcerative colitis, vasculitis, thyroid and adrenal cortex diseases, diabetes, high blood pressure, central nervous system disorders, and infectious and sexually transmitted diseases (through questions and file reviews); and
- No use of drugs that affect sexual function of the participants and their spouses, including psychoactive, cardiac and antihypertensive, thiazide diuretics, antidepressants, anticonvulsant, hypnotic, narcotic, hormonal, antihistamines, and anticancer drugs [1, 14, 15].

#### Inclusion criteria for health care providers

- Willingness and informed consent to participate in the study;
- At least 2 years of work experience

***Research environment:*** The participants will be accessed through comprehensive health service centers, gynecologic clinics of educational hospitals affiliated with Isfahan University of Medical Sciences, sexual health clinics, private offices of gynecologists and psychiatrists, psychology and counseling centers, and midwives’ offices.

*Data collection process* The researcher will initially obtain the necessary permits from the university and then refer to the desired centers and select the appropriate participants. Individual and semi-structured interviews will be recorded using an MP4. All observations of the non-verbal behaviors and interactions of the participants will be recorded using notes (field notes). The interviews will start with open questions and be guided through the participants’ open and interpretive answers. At the end of each interview, the resulting information will be immediately transcribed and analyzed simultaneously with data collection. Data collection will continue until data saturation when no new information is obtained during data analysis and coding.

*Data analysis* Data will be analyzed using the conventional qualitative content analysis method with Graneheim and Lundman’s approach [16]. After each interview, the recorded interview will be written verbatim, followed by frequently reading all the data to get an overview that leads to a semantic unit. The key concepts of the semantic units will be highlighted to extract the codes from which sub-categories and main categories will be subsequently extracted.

*Rigor and trustworthiness* This study will use various methods, including participant selection with maximum variety, allocation of enough time to collect data, in-depth interviews in different places and times, and combining several data collection methods, such as individual interviews and field notes, to improve the credibility of the findings. Participant review will also be used, along with a complete and continuous recording of the researcher’s decisions and activities, to ensure the dependability of the findings. The findings will be presented to several individuals with the same characteristics as absent participants in the study to judge the similarities between the research results and their experiences and increase the transferability. Besides, the texts of some extracted interviews, codes, and categories will be provided to faculty members who were not part of the research team but were familiar with qualitative research analysis to review the accuracy of the data coding process and increase confirmability.

### Phase II: designing the interventional program

#### Objectives

- Identifying strategies to improve the sexual function of women with endometriosis based on literature review;
- Prioritizing strategies to improve the sexual function of women with endometriosis (derived from a qualitative study and literature review) by experts;
- Drafting an interventional program to improve the sexual function of women with endometriosis and determining experts’ opinions about its content validity

#### Literature review

The existing knowledge will be searched in this section to confirm and complete the strategies adopted in the qualitative phase of the study to improve the sexual function of women with endometriosis and design an interventional program. The review will be conducted through Scopus, Web of Science, Cochrane Library, Google Scholar (as a search engine), SID, Magiran, Iranmedx, Medlib, and IranDoc. All studies published in Persian and English between 2000 and 2023 will be searched according to MeSh and keywords of sexual function, sexual dysfunction, sexual health, sexuality, sexual relations, sexual satisfaction, endometriosis, and women in both Persian and English.

#### Findings of literature review, qualitative study, and prioritizing solutions

In this section, the solutions obtained from literature review and qualitative research will be prioritized using the modified Delphi method [17], the opinions of experts, and decision matrix. The draft of solutions derived from the qualitative research and literature review will be changed into a matrix and provided to 10–15 experts (reproductive health specialists, gynecologists, and health education and promotion specialists) to prioritize the solutions in terms of importance, necessity, and feasibility. A score of 1 to 3 is considered for each dimension. The strategies will be prioritized after the decision matrix completion by experts. The draft interventional program will be designed according to the identified high-priority strategies.

#### Experts’ panel

A panel of experts, including reproductive health experts, gynecologists, health education and promotion experts, etc., will be held based on the interventional program to ask their opinions concerning the designed draft of program to improve the sexual function of women with endometriosis. The experts will be asked to comment on the design program for further modifications in the interventional program. Ultimately, the necessary modifications will be applied to the draft program to prepare the final version of the interventional program.

### Phase III: the implementation of the interventional program (quantitative study)

#### Objectives

- Determining the effect of the interventional program on improving the sexual function of women with endometriosis

*Type of the study* This phase will be conducted in the form of a two-group (intervention and control) quasi-experimental study at the individual level (women with endometriosis or women with endometriosis and their spouses) to determine the effectiveness of the interventional program to improve the sexual function of women with endometriosis.

*Research environment* The research environment will include comprehensive health service centers, gynecologic clinics of educational hospitals affiliated with Isfahan University of Medical Sciences, sexual health clinics, and private offices of gynecologists and midwives in Isfahan city.

*Research population* The research population will include all women with endometriosis and, if necessary, their spouses referring to the research environment during the data collection period.

*Research sample* The research sample will be women with endometriosis and, if necessary, their spouses who meet the inclusion criteria.

*Sample size and sampling method* The sample size will be calculated based on the results of previous studies and considering 95% confidence interval, 80% test power, S = 0.25–0.1, and 10% sample drop. Women with endometriosis who meet the inclusion criteria will be selected by convenience sampling.

#### Inclusion criteria

- No history of participation in training courses focused on sexual health education;
- Obtaining a score of < 28 from the Female Sexual Function Index (FSFI) standard questionnaire [18];
- Other inclusion criteria are similar to those in qualitative research

#### Exclusion criteria

- Diagnosis of other diseases in the women or their spouses during the program implementation and follow-up period that may affect their sexual function;
- Failure to regularly participate in the interventional program (failure to receive 50% intervention for any reason);
- Unwillingness to continue participation in the study;
- Incomplete questionnaires.

*Data collection methods* The data will be collected from the two groups using appropriate tools (such as the FSFI) in the pre- and post-intervention stages.

*The implementation method* The designed program will be implemented after the permission of the Ethical Committee of Isfahan University of Medical Sciences and the necessary arrangements with the centers. Participants will be selected through convenience sampling if they are willing to participate in the study and meet the inclusion criteria. Informed consent will be taken from all participants, after which the interventional program will be implemented, and both groups will complete the questionnaires within the pre- and post-intervention stages.

*Data analysis* The collected data will be analyzed using descriptive statistical methods (mean, standard deviation, minimum, and maximum), inferential statistics (paired t-test, chi-square, Fisher’s exact test, Wilcoxon and Mann–Whitney tests, and one-way ANOVA), and SPSS.20.

### Integration of the qualitative and quantitative data

The results of the quantitative and qualitative phases of the study will be mixed to present an interventional program focused on improving the sexual function of women with endometriosis.

### Ethical considerations

The Ethics Committee of the Isfahan University of Medical Sciences, Isfahan, Iran, approved the protocol of this study (Code number: IR.MUI.NUREMA.REC.1402.095). Informed consent, anonymity, confidentiality of information and the right to withdraw at any time will be observed. Also, the reasons for the study will be explained prior to each individual interview.

## Discussion

Sexual health is an essential aspect of the quality of life, while sexual dysfunction may decrease the quality of life and increase the occurrence of psychological, interpersonal, and marital problems. Thus, it is necessary to adopt a biopsychosocial approach for clinical care in female sexual function [9, 19]. The complex nature of endometriosis and its impact on the physical, psychological, and relational health of the patients (e.g., the sexual function of the partner) and the presence of other biological, psychological, and social variables necessitate a more comprehensive analysis of sexual health [6, 20]. Such a comprehensive treatment plan should be tailored to the needs and multifaceted problems of patients, while other issues such as emotional and social support, pain management, stress management, and psycho-sexual therapy should also be considered in addition to the surgical and pharmacological treatment [6, 21].

The current mixed-methods study will use a sequential exploratory approach to provide an interventional program compatible with the culture and conditions of Iran to improve the sexual function of women with endometriosis. This research will have two stages, with the results of the first stage leading to the second stage, facilitating a more comprehensive understanding of the phenomenon and the relationship among its various aspects [22–24]. When the quantitative or qualitative research methods alone are not enough to achieve the research objectives, a combination of the two methods will be taken into account [25]. Since the sexual function of women with endometriosis is multidimensional, and the factors affecting it cannot be predicted, a mixed-methods study using a combination of qualitative and quantitative research methods will be suitable. This research seeks to take an effective step in improving the sexual function of women with endometriosis and enhancing the quality of the couples’ sexual relations by designing and implementing an appropriate, indigenous, and culture-sensitive interventional program using a mixed method. If effective, this program can set the grounds for health policies and plans to strengthen the body of knowledge and improve the sexual health of women with endometriosis in the country.

## Data Availability

The datasets generated and/or analyzed during the current research are not publicly available as individual privacy could be compromised but are available from the corresponding author on reasonable request.

## Abbreviations

ART: Assisted reproductive techniques
FSFI: Female sexual function index
ANOVA: Analysis of variance
SPSS: Statistical package for the social sciences
